# Patient and Operator Centered Outcomes in Implant Dentistry: Comparison between Fully Digital and Conventional Workflow for Single Crown and Three-Unit Fixed-Bridge

**DOI:** 10.3390/ma13122781

**Published:** 2020-06-19

**Authors:** Paolo De Angelis, Paolo Francesco Manicone, Silvio De Angelis, Cristina Grippaudo, Giulio Gasparini, Margherita Giorgia Liguori, Francesca Camodeca, Giovan Battista Piccirillo, Viviana Desantis, Giuseppe D’Amato, Antonio D’Addona

**Affiliations:** 1 Division of Oral Surgery and Implantology, Department of Head, Neck, and Sensory Organs, Fondazione Policlinico Universitario A. Gemelli IRCCS—Università Cattolica del Sacro Cuore, 00168 Rome, Italy; paolofrancesco.manicone@unicatt.it (P.F.M.); cristina.grippaudo@unicatt.it (C.G.); marghegliguori@gmail.com (M.G.L.); francescacamodeca@gmail.com (F.C.); giovanbpiccirillo@gmail.com (G.B.P.); desantisviviana@gmail.com (V.D.); antoniodaddona@gmail.com (A.D.); 2 Private Practice, 63100 Ascoli Piceno, Italy; dr.silviodeangelis@gmail.com; 3 Oral and Maxillofacial Surgery Unit, Department of Head, Neck, and Sensory Organs, Fondazione Policlinico Universitario A. Gemelli IRCSS—Università Cattolica del Sacro Cuore, 00168 Rome, Italy; drgiuliogasparini@gmail.com; 4 Private Practice, 00168 Rome, Italy; dottgdamato@gmail.com

**Keywords:** digital dentistry, CAD/CAM, implant dentistry, prosthodontics

## Abstract

Background: Scientific information about the effects of implant therapy following a precise workflow and patient and operators’ preferences should be considered to choose which implant treatment protocol to use, and to achieve patient’s satisfaction and functional results. The aim of this study was to analyze implant rehabilitations with a fully digital workflow and compare this approach with a conventional one. Methods: This study comprises 64 patients treated with a fully digital approach and 58 patients treated using a conventional protocol. Patient and operator centered outcomes were assessed through two visual analogue scale (VAS) questionnaires. Results: The VAS questionnaire demonstrated better results for the digital workflow concerning anxiety, convenience, taste, nausea sensation, pain and breathing difficulties (*p* < 0.0001). The VAS questionnaire administered to the operators showed better scores for the digital approach in relation to anxiety, convenience, difficulties of the impression procedure and the workflow (*p* < 0.0001). A significant reduced mean time for the digital workflow as well as a reduced number of required visits were recorded. Conclusion: The analysis of a fully digital and a conventional protocol showed better results according to patient and operators’ preferences when a fully digital approach was used.

## 1. Introduction

Dentistry has changed significantly thanks to the introduction of new digital technologies and materials such as computer-aided design and computer-aided manufacturing (CAD/CAM), laser-sintering/melting and 3D printing in the daily clinical practice [1]. Nowadays, studies about prosthetic rehabilitation using dental implants are based on the assessment of clinical parameters. Longevity can be assessed through the survival rate while success rate, probing depth (PD), bleeding on probing (BOP) and other radiographical outcomes are focused on the evaluation of the technical and biological effects of the therapy [2,3,4,5]. Patient- and operator-centered outcomes concerning implant treatment protocols are rare and mostly focused on the patient’s satisfaction with the functional results obtained at the end of the treatment [6]. Scientific information about the effects of an implant therapy following a precise workflow as well as patient and operators’ preferences should be considered to choose adequately which protocol to use.

According to the 9th edition of The Glossary of Prosthodontics Terms, “impression” is defined as “a negative likeness or copy in reverse of the surface of an object; an imprint of the teeth and adjacent structures for use in dentistry” [7]. There is some variability in impressions and the resulting master casts, depending on the technique and material used by the operator [8].

In the so-called “conventional” technique, a cast is produced after an impression has been made with a tray filled with an impression material. In the digital system, the intraoral condition has been digitally recorded using an intraoral 3D acquisition device, and the acquired information enables the computer to generate a virtual model. The digital impression technique has many advantages, such as (1) improvement of patient acceptance, (2) reduction in the distortion of impression materials, (3) the possibility of providing improved 3D previsualization of the abutments, and (4) potential cost- and time-effectiveness [9,10,11].

Today, patients expect a successful clinical outcome obtained through more convenience-oriented treatment protocols. With the implementation of intraoral scanning (IOS), patients are prevented from harm during classical impression taking procedures due to suffocation hazard, gagging, and taste irritation [9,12].

CAD/CAM technology changed dentistry, affecting both dental laboratories and dental offices deeply, especially in the fields of prosthodontics and restorative dentistry [12,13,14]. However, the majority of studies about digital dentistry have focused mostly on developing new and more sophisticated protocols, assessing the accuracy and precision of intraoral scanners and dental milling machines as well as evaluating the clinical and aesthetic outcomes of the new materials applied to the digital dentistry [15]. Moreover, the CAD/CAM technology has also been applied in the development of CAD/CAM titanium bars as a restorative solution for the edentulous and partially edentulous arch. They can be customized to achieve a better fit on the tooth and on the surrounding soft tissues [16]. According to Scarano et al., this leads to a simplification in terms of prosthetic workflow thanks to the high precision of this technology [17]. Moeller et al. suggest that CAD/CAM titanium bars can also be used in case of poorly positioned implants [18]. The change of a well-established protocol requires a scientific validation which is paramount to understand the impact of this approach on the patients as well as on the operators’ daily practice which can be represented by a crown or bridge work on implants. Few studies have investigated a fully digital workflow on implant restorations in order to scientifically validate this approach, considering patient and operators’ perceptions as well as time, efficiency and costs [19].

The aim of this retrospective study was to analyze implant rehabilitations with a fully digital workflow and compare this approach with a conventional one. The first null hypothesis was that there is no difference in patient- and operator-centered outcomes between the conventional and digital workflow when applied to single restorations and three-unit fixed bridges on implants. The second null hypothesis was that there is no difference in time efficiency between conventional and digital protocols.

## 2. Materials and Methods

A multicenter retrospective clinical study was conducted selecting edentulous patients needing implant restorations in the mandibular or maxillary premolar and molar sites treated between 1 January 2016 and 1 January 2018. This study comprises 64 patients who had been treated consecutively with a screw-retained monolithic single crown or a screw-retained monolithic three-unit fixed bridge with a fully digital approach including a digital impression, and 58 patients who received a screw-retained monolithic single crown or a screw-retained monolithic three-unit fixed bridge using a conventional impression technique. All the implants installed were dental implants of the Straumann Implant System. All patients were treated in two private practices by two trained clinicians (P.D.A., S.D.A.) who performed both the surgical and prosthetic phases of the implant rehabilitation. All prosthetic restorations were performed by the same laboratory technician using a standardized protocol.

Eligibility criteria stipulated that patients must have been edentulous for a minimum of 4 months, must have a general health showing no contraindications for implant treatment, must be at least 20 years old and must have not yet received implant restorations. In order to be included, patients also had to have good oral hygiene before the treatment, and FMPS (full mouth plaque score) and FMBS (full mouth bleeding score) cut-off was set at 15%. 

Patients were excluded if they had signs or symptoms of temporomandibular disorders, bruxism or clenching, if they were suffering from uncontrolled systemic conditions or if they presented an American Society of Anesthesiologists physical status classification of IV. From an initial sample of 147 eligible patients, 12 patients moved out of the city and 13 patients were not available or did not want to participate in the retrospective study, leaving a total of 122 participants. This research was conducted in private practice, and the medical devices evaluated had already been approved for clinical use. All investigations reported were carried out in accordance with the 1975 Helsinki Declaration, as revised in 2013 for ethical approval. All participants provided written informed consent after receiving explanations on study objectives and procedures. Because of the retrospective nature of this study, it was granted an exemption in writing by the local ethics committee.

Primary outcomes were patient- and operator-centered outcomes. Patient- and operator-centered outcomes were assessed through two different visual analogue scale (VAS) questionnaires as previously performed by Joda et al. containing, respectively, 9 and 7 self-developed questions about each phase of the treatment which were administered at the end of each phase. Answers were recorded with a hash mark on a not numeric 100 mm line [2]. Questions focused on the treatment time, on the self-perception of the applied impression protocol in terms of general convenience, anxiety, taste, nausea sensation and possible pain sensation. The questionnaires were administered as part of a survey which aimed to obtain information about the dental practices.

Secondary outcomes were time-efficiency by assessing workflow duration and number of appointments needed to complete the rehabilitation. The treatment phases considered were impression and prosthetic placement.

Time was recorded in minutes. All the parameters were recorded in the clinical chart by the same two operators (M.G.L., F.C.) not involved in the treatment after each phase was completed. Detailed time assessment was separately defined for digital and conventional procedures as shown in Tables 3 and 4.

### 2.1. Clinical Procedures

**Figure 1:**
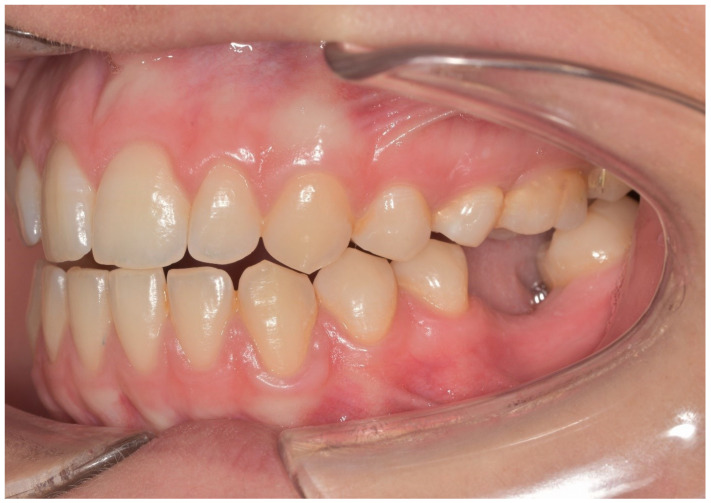
Preoperative evaluation of the patient.

**Figure 2:**
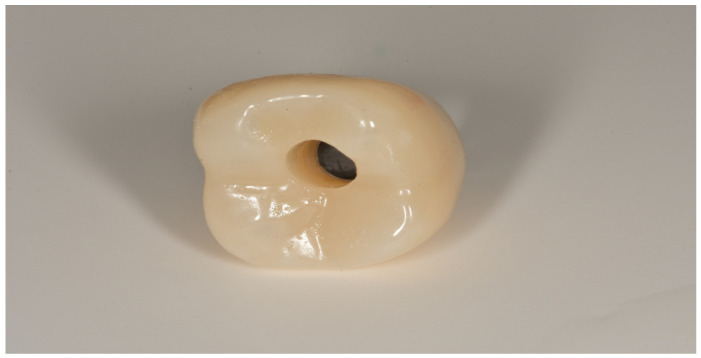
Occlusal view of the monolithic CAD/CAM lithium disilicate crown with the screw access hole.

**Figure 3:**
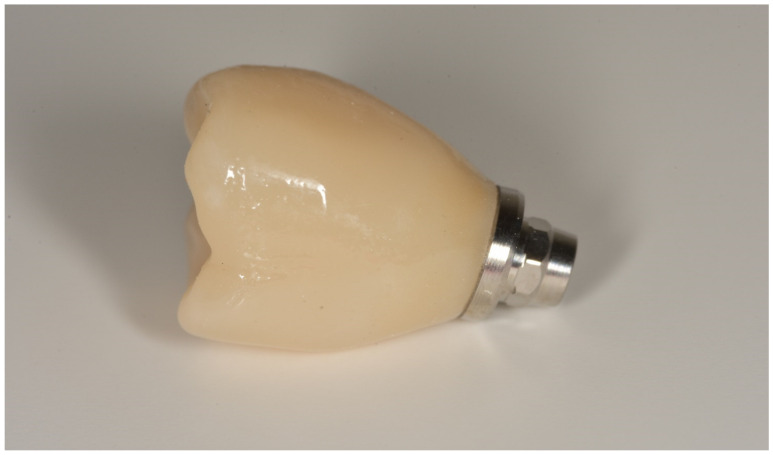
Lateral view of the monolithic CAD/CAM lithium disilicate crown luted to the screw-retained abutment.

**Figure 4:**
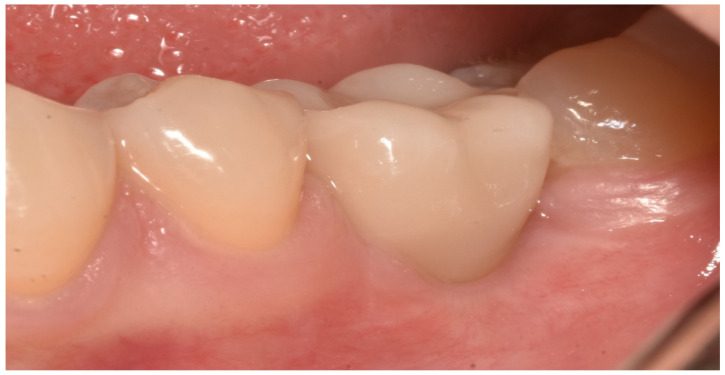
Prosthetic placement of the crown.

In both groups, the fixtures were inserted (Bone Level or Tissue Level SLA Roxolid, Straumann Group) under local anesthesia, following guidelines for the Straumann Implant System (Figure 1). In the full digital group, after a healing period of 3 months, the prosthetic rehabilitation started taking a digital impression using CEREC AC Omnicam (Sirona Dental System, Long Island City, N.Y., U.S.A.) and an implant-specific scan body which provided the 3D registration of the implant. The dental technician used CEREC inLab (Sirona Dental System) to design and fabricate each single crown. Prefabricated abutments were used (Variobase, Straumann Implant System, Basel, Switzerland). Full-contoured acrylic resin prostheses were made first and tried-in intraorally according to the clinical situation. Monolithic CAD/CAM lithium disilicate crowns (IPS e.max CAD Abutment Solutions, Ivoclar Vivadent, Schaan, Liechtenstein) were milled using inLab MC XL (Sirona Dental System) and crystallized using Programat XP1 (Ivoclar Vivadent) (Figure 2 and Figure 3). Fixed bridges were designed by the dental technician and full-contoured acrylic resin prostheses were made first and tried-in intraorally. Monolithic CAD/CAM zirconia three-unit fixed bridges (inCoris TZI, Sirona Dental System) were milled and sintered. Prefabricated metal substructures (screw-retained abutments and copings for screw-retained abutments) were used for fabrication of one-piece screw-retained fixed dental prostheses. All the crowns and bridges were bonded using an adhesive luting composite (Multilink Hybrid Abutment, Ivoclar, Schaan, Liechtenstein) and finally polished. The screw access holes were filled with a polytetrafluoroethylene tape and a composite resin (Figure 4).

In the conventional group, after a healing period of three months, the prosthetic rehabilitation started taking a conventional impression with alginate impression material (Xantalgin, Mitsui Chemical Group, Tokyo, Japan) and stock trays. A custom tray was made for the final impression and the open tray technique using pickups and polyether impression material (Impregum Penta Soft Quick Step MB, 3M ESPE) was performed according to the manufacturer’s guidelines. Starting from a conventional master model, scanning procedures were performed to obtain with an appropriate protocol the same type of restorations used in the full digital group. Full-contoured acrylic resin prostheses were made first and tried-in intraorally according to the clinical situation Monolithic CAD/CAM lithium disilicate crowns (IPS e.max CAD Abutment Solutions, Ivoclar Vivadent), and monolithic CAD/CAM zirconia three-unit fixed bridges (inCoris TZI, Sirona Dental System) were performed. All the crowns and bridges were bonded to the prefabricated metal substructures using an adhesive luting composite (Multilink Hybrid Abutment, Ivoclar) to create a one-piece screw-retained restoration and finally polished. The screw access holes were filled with a polytetrafluoroethylene tape and a composite resin.

### 2.2. Statistical Analysis

Values were expressed as mean and SD for continuous variables or absolute frequency and percentages for categorical variables. Continuous variables were compared with parametric (Student’s *t*-test). One-way analysis of variance was used to testify an association between the scores of the questionnaire and the detected anamnestic data. All statistical analyses were carried out using STATA14 for Windows software with a two tailed *P* value of 0.05 used as a threshold for significance.

## 3. Results

A total of 122 patients (81 females and 41 males; mean age ± standard deviation: 58.3 ± 6.9 years, range: 37–64 years) were enrolled for the study. All the patients were partially edentulous. On the whole, both protocols allowed us to reach excellent results with no complications recorded during the procedures. In the digital group, 41 (64%) patients received a single crown restoration while 23 (36%) patients had a three-unit fixed bridge. In the conventional group, 33 (57%) patients received a single crown restoration while 25 (43%) patients had a three-unit fixed bridge.

**Figure 5:**
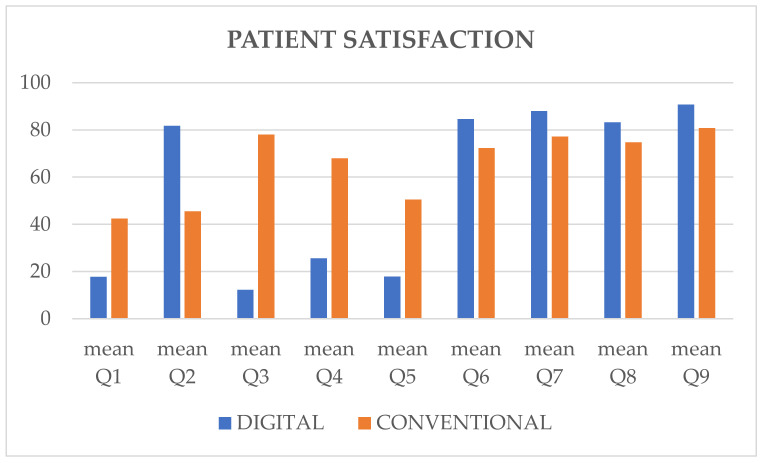
Mean scores of patient-centered outcomes with digital and conventional protocol.

Table 1 shows the mean results from the visual analogue scale questionnaires administered to patients who analyzes treatment time, patients’ subjective convenience level, anxiety, bad oral taste, nausea sensation, possible pain sensation and breathing difficulties during impression taking. In general, there were statistically significant differences (*p* < 0.0001), always favoring the digital protocol over the conventional one (Figure 5). In addition, there was a statistically significant difference between the two protocols regarding patients’ opinion of the treatment protocol, the operator and the dental practice (Table 1), showing a preference for the digital approach. No statistically significant associations were observed between each question administered and the variables of gender and age (*p* > 0.05).

**Figure 6:**
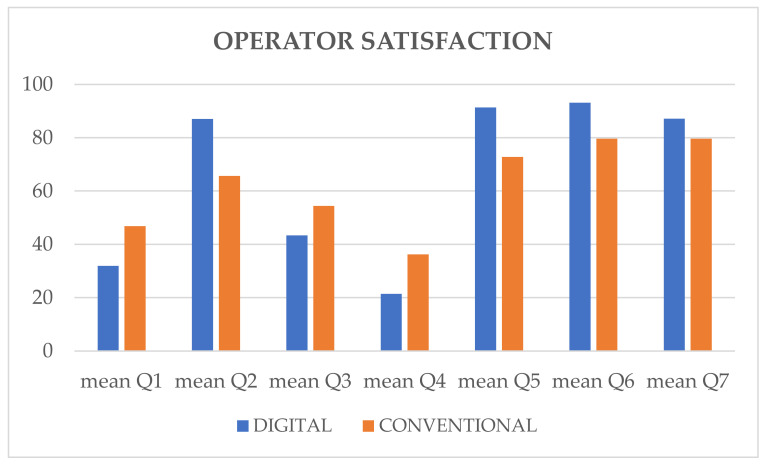
Mean scores of operator-centered outcomes with digital and conventional protocol.

The visual analogue scale questionnaire administered to the operators showed better mean scores for the digital approach in relation to anxiety, convenience, difficulties of the impression procedure and the workflow as seen in Table 2. The operators also reported that they perceive a better response from the patients treated following the digital protocol as seen in Table 2 (Figure 6). All the differences were statistically significant (*p* < 0.0001). Thus, the first null hypothesis of the study was rejected. All patients would select the digital approach for future implant restorations, while 15 (26%) patients in the conventional group would change this approach as seen in Table 1. 

The analysis of the secondary outcomes revealed a significant reduced mean time of the digital workflow (*p* < 0.0001) as well as a reduced number of required visits as seen in Table 3 and Table 4. The second null hypothesis of the study was rejected.

## 4. Discussion

The main purpose of the study was to focus on the patient and operators’ perceptions regarding implant rehabilitations with a fully digital workflow and compare this approach with a conventional one. CAD/CAM procedures and digital workflow are now common in daily clinical practice because of their excellent results in terms of use and quality obtained. A retrospective clinical study by De Angelis et al. reported that monolithic lithium disilicate and zirconia screw-retained single crowns fabricated using CAD/CAM and a fully digital workflow proved to be reliable and valuable clinical options for the implant-supported restoration of a posterior missing tooth [20].

In the present study, the digital workflow results supported the preferred protocol when compared with the conventional one for single and bridge work on implants. Thus, the null hypothesis of the study was rejected. 

Participants in our study included patients with no previous experience of implant restorations to avoid bias caused by previous experience, and the treatment was performed in a clinical setting by two experienced prosthodontists. All the parameters were assessed by the same assistant not involved in the treatment to achieve objective results. Furthermore, the evaluations were performed at the end of each phase to avoid the possibility of erasing from memory the effects of the procedures.

In the early phase of the prosthetic rehabilitation, thanks to the intraoral scanning, it was possible to reach a better level of comfort using digital impressions, which were preferred by the patients, even taking into consideration the bad taste related to the impression materials, the nausea sensation and the breathing difficulties caused by the procedure. Gherlone et al. reported that digital oral impression is able to eliminate several errors that can occur in the conventional method, such as the inaccurate reproduction of the preparation margins, the possible occurrence of lack of material in key areas and the presence of debris [21]. In addition, a study by Joda et al. used the VAS scale to evaluate the patient’s opinion of the digital impression compared to the conventional one [2]. They reported that, even if both workflows were clinically successful, the digital workflow was significantly accepted as the most preferred and time-effective implant impression procedure compared to the conventional technique with regard to the patients’ perception and satisfaction. Wismeijer et al. reported similar results investigating the effects of the digital impression on patients [22]. There were also recorded differences in the time duration when comparing the digital procedure with the conventional one, and this significative difference was perceived by the patients who were able to easily appreciate it. This finding was confirmed by Yuzbasioglu et al. and Lee et al., who assert that the major advantage of digital impression is to reduce the chair time [11,23].

In the present study, the treatment acceptance was higher in the digital workflow, and the opinions of the patients in relation to the operators and to the dental practice in general were significantly and positively influenced by the use of digital technologies. Based on these results, patients undergoing prosthetic rehabilitation with a digital protocol may benefit from more comfort and report a more pleasant experience.

The workflow was also evaluated by the operators, who mostly appreciated the reduction of the treatment time and the simplification of the therapy without encountering difficulties in any of the phases of the therapy. In a study by Lee et al., the level of difficulty judged by the operator was significantly lower if the digital procedures were used when compared with conventional implant impressions [23]. Furthermore, the operator perceived a better patient response when digital technologies were applied in explanation and treatment (to treat the patient and explain the therapy). However, a higher difficulty was recorded for multiunit fixed bridges outlining that digital technologies require an adequate learning curve, especially in more complex cases.

The main advantages of CAD/CAM technologies are the possibilities of shortening clinical treatment time and reducing costs without affecting the overall quality. Furthermore, physical models are unnecessary, making it easier and faster to communicate with the dental lab [24,25].

Time efficiency is a parameter rarely analyzed in the literature [26]. Our results showed a higher efficiency using the digital workflow thanks to the simplification of the treatment and the use of monolithic materials with excellent biomechanics and aesthetic features. The digital protocol tends to reduce the number of visits, thus increasing treatment effectiveness [26]. The differences recorded were greater for bridge works. Furthermore, with the fully digital protocol, the number of required visits to complete the prosthetic rehabilitation can be shortened to a single appointment, without affecting the quality and the aesthetic result, if the patient chooses to undergo a longer visit in order to end the treatment in one session. Thus, the second null hypothesis of the study was rejected. The growing success of the digital workflow is most likely also due to the development of monolithic materials to be milled using CAD/CAM technology, as they have demonstrated excellent biomechanical, biological and biomimetic properties [20,27]. Zirconia is a crystalline dioxide of zirconium that is the strongest ceramic used in restorative dentistry for the fabrication of single and multiple unit restorations (monolithic or veneered) in the anterior and posterior region. Monolithic zirconia has shown excellent mechanical properties such as the high flexural strength (900‒1200 MPa) and the unique crack inhibitory property [28,29,30,31]. On the other hand, lithium disilicate is a glass-ceramic used for the fabrication of monolithic or veneered single restorations which is used in the posterior region only for single crowns and three-unit restorations with the second premolar as terminal abutment. Monolithic lithium disilicate crowns have shown excellent light-optical and aesthetic properties as well as adequate mechanical properties (mean flexural strength of 530 MPa), which made it a material of choice for single restorations both on the anterior and posterior regions [32,33]. 

Studies considering patient- and operator-centered outcomes are rare in the literature and have focused on the assessment of the best prosthetic solution for each type of edentulism, considering the functional results achievable [34]. Thus, patient- and operator-centered outcomes are scarcely used in analyzing one workflow adopted to achieve a certain prosthetic rehabilitation, even if they should be considered before choosing one protocol over the others. However, in the present study, patient and operators’ preferences were for the digital approach. In both groups, it was possible to achieve the planned prosthetic rehabilitation. It should be remembered that, in this study, patients with adequate bone volume in which stock devices were indicated have been selected [35]. However, the present study has limitations, in particular the retrospective nature, the small and strictly selected sample and the short term follow-up. The strong points are the surgical and prosthetic protocols that were strictly followed by the same clinicians, the use of the same intraoral scanner and the digital impression technique [29,31]. Therefore, to confirm the results obtained, further randomized large-scale studies with a longer follow-up are necessary. Furthermore, this study addressed only a few patient and operator outcomes, and for this reason, other aspects need further investigations to fully understand the effects of each protocol as well as the feasibility for a non-experienced operator.

## 5. Conclusions

Dentistry has evolved significantly with the introduction of new technologies and protocols, so patient and operators’ preferences should be taken into consideration to choose the most appropriate one. Based on the results of this prospective clinical study, the analysis of a fully digital and a conventional protocol showed better results according to patient and operators’ preferences for implant-supported single crown and three-unit fixed bridges when a fully digital approach was used. The main differences are caused by a better compliance from patients due to clinical parameters, such as minor anxiety, nausea sensation, pain and breathing difficulties, and a better perception from operators, in relation to other parameters, like anxiety, convenience, difficulties of impression procedure and workflow. 

## Figures and Tables

**Table 1:** Questions on patient satisfaction with digital and conventional protocol and mean scores of the results.

Questions	Conventional Protocol			Digital Protocol			*p* Values
	Mean ± SD	Median	Range	Mean ± SD	Median	Range	
**Q1) How high was your anxiety after the informative consultation and before the beginning of the treatment? ^1^**	42.4 ± 15.6	43	19–65	17.7 ± 10.7	17	1–36	*p* < 0.0001
**Q2) How convenient was the impression procedure for you? ^2^**	45.5 ± 8.8	47	30–60	81.8 ± 5.6	82	73-93	*p* < 0.0001
**Q3) Was there a bad oral taste and/or after the impression procedure? ^3^**	78.1 ± 3	78	73–83	12.2 ± 4.1	12	7–21	*p* < 0.0001
**Q4) Did you experience a sensation of nausea during the impression procedure? ^4^**	68 ± 5.6	67	59–77	25.6 ± 8	26	13–40	*p* < 0.0001
**Q5) Did you experience pain or breathing difficulties during the impression procedure? ^5^**	50.5 ± 8.7	52	37–64	17.8 ± 5.3	18	9–25	*p* < 0.0001
**Q6) What is your opinion on the treatment time required to achieve prosthetic rehabilitation? ^6^**	72.3 ± 6.2	72	61–83	84.8 ± 6.7	85	23–73	*p* < 0.0001
**Q7) What is your general opinion on the protocol of treatment? ^7^**	77.2 ± 7.5	78	65–89	88 ± 6	87	79–99	*p* < 0.0001
**Q8) What is your opinion of the operators? ^8^**	74.8 ± 5.5	75	66–84	83.3 ± 4.2	84	77–91	*p* < 0.0001
**Q9) What is your opinion of the dental practice? ^9^**	80.8 ± 5.1	80	73–89	90.8 ± 5.3	91	82–99	*p* < 0.0001

^1^ VAS: no anxiety 0–100 top level of anxiety, ^2^ VAS: unsatisfactory 0–100 excellent, ^3^ VAS: no bad taste 0–100 top level of bad taste, ^4^ VAS: no nausea 0–100 top level of nausea, ^5^ VAS: no pain 0–100 top level of pain, ^6^ VAS: unsatisfactory 0–100 excellent, ^7^ VAS: unsatisfactory 0–100 excellent, ^8^ VAS: unsatisfactory 0–100 excellent, ^9^ VAS: unsatisfactory 0–100 excellent.

**Table 2:** Questions on operator satisfaction with digital and conventional protocol and mean scores of the results.

Questions	Conventional Protocol			Digital Protocol			*p* Values
	Mean ± SD	Median	Range	Mean ± SD	Median	Range	
**Q1) How high was your anxiety before the beginning of the treatment? ^1^**	46.8 ± 10.8	48	26–66	31.9 ± 7	32	20–44	*p* < 0.0001
**Q2) How convenient was the impression procedure for you? ^2^**	65.7 ± 6.4	66	55–77	87 ± 5.9	86	78–98	*p* < 0.0001
**Q3) How difficult was the impression procedure for you? ^3^**	54.4 ± 8.8	56	39–69	43.4 ± 7.8	43	31–58	*p* < 0.0001
**Q4) How difficult was the workflow for you? ^4^**	36.2 ± 11	35	21–55	21.4 ± 4.7	21	14–29	*p* < 0.0001
**Q5) What is your opinion on the treatment time required to achieve prosthetic rehabilitation? ^5^**	72.8 ± 6.8	74	61–83	91.3 ± 5	91	82–100	*p* < 0.0001
**Q6) What is your opinion of the protocol of treatment? ^6^**	79.6 ± 7.3	79	69–93	93.1 ± 4.2	93	87–100	*p* < 0.0001
**Q7) What is your opinion of the patient responses to the protocol? ^7^**	79.6 ± 3.9	79	73–86	87.1 ± 5.8	87	77–97	*p* < 0.0001

^1^ VAS: no anxiety 0–100 top level of anxiety, ^2^ VAS: unsatisfactory 0–100 excellent, ^3^ VAS: no 0–100 top level of bad taste, ^4^ VAS: no difficulties 0–100 top level of difficulty, ^5^ VAS: unsatisfactory 0–100 excellent, ^6^ VAS: unsatisfactory 0–100 excellent, ^7^ VAS: unsatisfactory 0–100 excellent.

**Table 3:** Time efficiency (conventional protocol).

Treatment Phase	Minutes (min) Mean ± SD (min)	
	Single Crown	Three-Unit Bridge
Alginate impression	6 ± 1	6 ± 1
Impregum polyether impression	20 ± 2	23 ± 2
Try in monolithic restoration	17 ± 4	20 ± 6
Prosthetic placement	14 ± 2	15 ± 4
**Number of appointments**	4	4

**Table 4:** Time efficiency (digital protocol).

Treatment Phase	Minutes (min) Mean ± SD (min)	
	Single Crown	Three-Unit Bridge
Digital impression	12 ± 2	15 ± 2
Try in monolithic restoration	16 ± 4	21 ± 5
Prosthetic placement	12 ± 5	14 ± 6
**Number of Appointements**	3	3
